# Dependency, Mortality, Invisibility: Linking Childhood Disability with Life Course Health Outcomes

**DOI:** 10.1093/geroni/igab046.3072

**Published:** 2021-12-17

**Authors:** Jessica Hoyle, James Laditka, Sarah Laditka

**Affiliations:** University of North Carolina at Charlotte, Charlotte, North Carolina, United States

## Abstract

People who experienced disability in childhood are living longer. It is not clear if longer lives indicate better health and less dependency, or if longer life is accompanied by increased dependency. We addressed that question by studying the joint dynamics of mortality and dependency. This population is “invisible” in most national surveys, which do not ask about childhood disability. We evaluated special education history as an indicator of childhood disability, and used that indicator to estimate dependency and life expectancy throughout adult life. Data: Panel Study of Income Dynamics and the Health and Retirement Study (n=20,563). Activities of daily living (ADLs), instrumental ADLs, and cognition defined five functioning levels including dependency and death. Multinomial logistic Markov models estimated probabilities for transitioning among the levels, with or without a history of childhood disability, adjusted for demographics. We used the probabilities in microsimulations, creating large populations of completed lives, identifying dependency at each age for each individual. Analysis showed special education history was a valid indicator of childhood disability; 13% had such history. With parent education less than high school, remaining life at age 20 was 46.0 years for people with that history, 58.3 for others; corresponding results with parent’s bachelor’s degree: 48.3 and 60.7 (p < 0.05). Corresponding population percentages dependent 5+ years were: 15.2% and 3.8%, 13.1% and 3.8% (all p<0.05). Special education history can indicate childhood disability. People with that history had significantly more dependency than others, and shorter lives. Accommodations and interventions can improve their health and functioning.

